# Detecting Diabetic Ketoacidosis with Infection: Combating a Life-Threatening Emergency with Practical Diagnostic Tools

**DOI:** 10.3390/diagnostics13142441

**Published:** 2023-07-21

**Authors:** Rahnuma Ahmad, Mahendra Narwaria, Arya Singh, Santosh Kumar, Mainul Haque

**Affiliations:** 1 Department of Physiology, Medical College for Women and Hospital, Dhaka 1230, Bangladesh; 2 Asian Bariatrics Plus Hospital, V Wing-Mondeal Business Park, S G Highways, Ahmedabad 380054, India; 3 Department of Periodontology, Karnavati School of Dentistry, Karnavati University, Gandhinagar 382422, India; 4 Unit of Pharmacology, Faculty of Medicine and Defence Health, Universiti Pertahanan Nasional Malaysia (National Defence University of Malaysia), Kuala Lumpur 57000, Malaysia; 5 Department of Scientific Research Center (KSRC), Karnavati School of Dentistry, Karnavati University, Gandhinagar 382422, India

**Keywords:** diabetic ketoacidosis, bacterial infection, inflammation, biomarkers, sepsis, early diagnosis, emergency

## Abstract

Background: Diabetic ketoacidosis (DKA) is a life-threatening acute complication of diabetes mellitus and can lead to patient demise if not immediately treated. From the recent literature, the diabetic ketoacidosis mortality rate, depending on age, is 2–5%. Insulin discontinuation and infection remain the two most common triggers for diabetic ketoacidosis. About 50% of cases of ketoacidosis result from bacterial infections like urinary tract infections and pneumonia. It is also important to diagnose the presence of infection in diabetic ketoacidosis patients to prevent the excessive use of antibiotics, which may lead to antibiotic resistance. Although performing bacterial culture is confirmatory for the presence or absence of bacterial infection, the time required to obtain the result is long. At the same time, emergency treatment needs to be started as early as possible. Methods: This narrative review examines various septic markers to identify the appropriate tools for diagnosis and to distinguish between diabetic ketoacidosis with and without infection. Electronic databases were searched using the Google engine with the keywords “Diabetes Mellitus”, “Diabetic Ketoacidosis”, “Infection with Diabetic Ketoacidosis”, “biomarkers for infection in Diabetic Ketoacidosis”, “Procalcitonin”, “Inflammatory cytokines in DKA”, “Lactic acidosis in DKA”, and “White blood cell in infection in DKA”. Results: This narrative review article presents the options for diagnosis and also aims to create awareness regarding the gravity of diabetic ketoacidosis with infection and emphasizes the importance of early diagnosis for appropriate management. Diabetes mellitus is a clinical condition that may lead to several acute and chronic complications. Acute diabetic ketoacidosis is a life-threatening condition in which an excess production of ketone bodies results in acidosis and hypovolemia. Infection is one of the most common triggers of diabetic ketoacidosis. When bacterial infection is present along with diabetic ketoacidosis, the mortality rate is even higher than for patients with diabetic ketoacidosis without infection. The symptoms and biomarkers of diabetic ketoacidosis are similar to that of infection, like fever, C reactive protein, and white blood cell count, since both create an environment of systemic inflammation. It is also essential to distinguish between the presence and absence of bacterial infection to ensure the appropriate use of antibiotics and prevent antimicrobial resistance. A bacterial culture report is confirmatory for the existence of bacterial infection, but this may take up to 24 h. Diagnosis needs to be performed approximately in the emergency room upon admission since there is a need for immediate management. Therefore, researching the possible diagnostic tools for the presence of infection in diabetic ketoacidosis patients is of great importance. Several of such biomarkers have been discussed in this research work.

## 1. Introduction

Diabetic ketoacidosis (DKA) is a common diabetic complication associated with patient morbidity and mortality [1]. Although there has been much progress in the development of monitoring methods, disease awareness, and hypoglycemic medication, in the past ten years, there has been a consistent rise in mortality resulting from DKA and patient hospitalization [2]. The mortality rate due to DKA has been noted to be 2–5% in recent studies [3]. The most common DKA triggers are insulin therapy discontinuation and infections [4]. A study carried out in China reported that DKA was typically caused by infection (40.1%), followed by hypoglycemic agent discontinuation (16.8%) and idiopathic reasons (36.9%) [5]. Infection (the most common cause of DKA) is related directly to the risk of demise [4].

Infections due to bacteria that commonly lead to DKA and account for about 50% of all patients with DKA, are urinary tract infections and pneumonia [6]. Bacterial infection in DKA raises hospital stay duration and rate of mortality [7]. Such infections must be detected early and treated with proper antibiotics to improve outcomes for individuals suffering from DKA [4]. Infection diagnosis is typically based on symptoms and secretion culture. Reports of culture take about 24 to 48 h to produce [8]. An early diagnosis of infections may be challenging since specific symptoms of DKA are like that of infection, like fever, a raised leukocyte count, neutrophil percentage, C reactive protein (CRP), and procalcitonin (PCT) [9]. This may lead to overtreatment with antibiotics and the development of antibiotic resistance. It is therefore a time-demanding matter to search for effective and rapid diagnostic tools for the early detection of infection to prevent progression to life-threatening conditions.

## 2. Objectives of the Study

Diabetic patients suffer both acute and chronic complications [10]. Diabetic ketoacidosis is a life-threatening complication that requires immediate diagnosis and management to attempt the prevention of death [11]. There are several biomarkers present for the diagnosis of DKA [12,13]. The presence of infection may further worsen this life-threatening condition, the diagnosis of which is also vital to decide upon the need to administer antibiotics [4]. Since the injudicious use of antibiotics can lead to microbial resistance in these patients, it is of significance to build awareness regarding the various diagnostic tools that may aid in immediate diagnosis and distinguishing between DKA with and without infection [14]. This study aims to shed light on the possible diagnostic tools that may be utilized in the emergency department of hospitals upon the admission of patients with DKA and infection.

## 3. Materials and Methods

This narrative review focuses on identifying various diagnostic tools that can be used to diagnose and distinguish between DKA with and without infection. The review also attempts to narrate the possible mechanisms that can lead to the rise in these markers where applicable. The study was performed between May and June 2023. The search used electronic databases like Google Scholar, PubMed, Google Search Engine, Web of Science, and Science Direct. Additional articles were found from the list of references of the identified articles on the related topics. Keywords used for retrieving papers were “Diabetes Mellitus”, “Diabetic Ketoacidosis”, “Infection with Diabetic Ketoacidosis”, “Biomarkers for infection in Diabetic Ketoacidosis”, “Procalcitonin”, “Inflammatory cytokines in DKA”, “Lactic acidosis in DKA”, and “White blood cell in infection in DKA”. These keywords were typed into the electronic search platforms like Google search engine, Pubmed, and Science Direct, which then displayed articles related to the keywords. These articles were read, and suitable ones were included in the study. The literature and articles dating before 2000 and in foreign languages other than English were not included in the study. Hand-searching of the articles was performed prior to inclusion in this research work.

## 4. Biochemical Criteria for DKA

**Figure 1:**
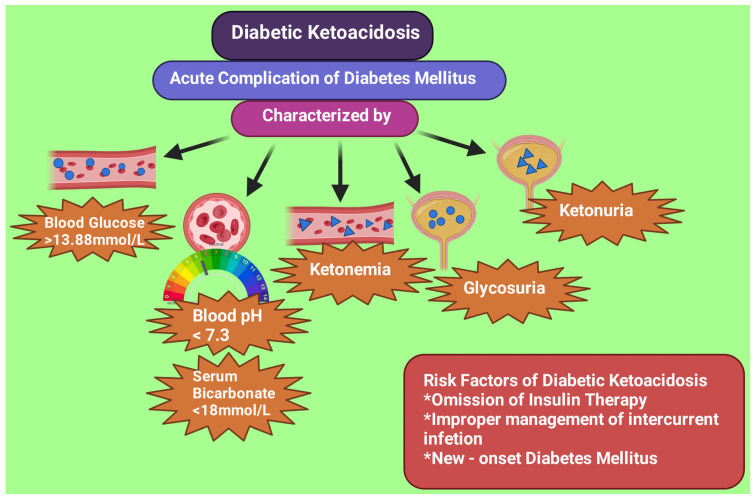
Biochemical characteristics and risk factors of diabetic ketoacidosis. This figure has been drawn with the premium version of BioRender (https://biorender.com/ accessed on 28 June 2023) with license number TD25LTFJ4F. Image credit: Rahnuma Ahmad.

DKA occurs in individuals with diabetes mellitus who omitted insulin or had improper intercurrent illness management or subjects having new-onset diabetes [15]. The ketoacidosis biochemical criteria include: (a) pH less than 7.3, (b) serum bicarbonate less than 18 mmol/L, (c) plasma glucose level more than 13.88 mmol/L, and (d) a raised ketone level in serum and the presence of ketone in urine [16] (Figure 1). The three stages of ketoacidosis severity based on levels of pH and bicarbonate include: (a) a mild stage with pH less than 7.3 or bicarbonate less than 18 mmol/L, (b) a moderate stage with pH less than 7.2 or bicarbonate less than 10 mmol/L, and (c) a severe stage with pH less than 7.1 or bicarbonate less than 5 mmol/L [16,17].

## 5. DKA Mimicking Bacterial Infection

**Figure 2:**
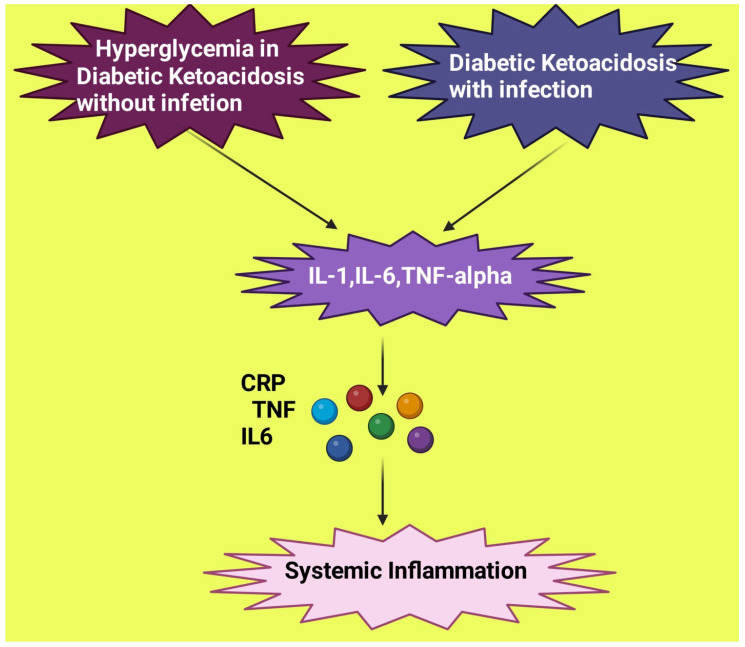
Similar inflammatory response exhibited by diabetic ketoacidosis with and without infection. IL: Interleukin; TNF: tumor necrosis factor; CRP: C reactive protein. This figure has been drawn with the premium version of BioRender (https://biorender.com/ accessed on 23 June 2023) with license number RS25JEG08R. Image credit: Rahnuma Ahmad.

Since DKA symptoms often have similarities to infection, it is difficult to differentiate between an inflammatory response due to sepsis and non-sepsis (Figure 2). The formation and secretion of cytokines are stimulated via hyperglycemia. There is an elevation of plasma CRP, interleukin-1 β, interleukin-6, and TNF-α in diabetic individuals without infection [18]. A study by Hoffman noted a significant rise in all cytokines in patients with DKA compared to those without DKA [19]. However, the study reported that neutrophil percentage, leukocyte count, and CRP were more in the case of DKA with infection when compared to patients without infection.

## 6. Diagnostic Tools for DKA

### 6.1. The Link between Serum Lactate and Severity of DKA

Diabetic ketoacidosis is a type of metabolic acidosis with a high anion gap associated with hyperglycemia, high levels of ketone, and acidosis that may, in severe cases, lead to the individual’s demise [20]. Anaerobic metabolism in the body produces lactate primarily in muscle tissue by pyruvate reduction with the help of lactate dehydrogenase enzyme [21]. The lactate levels remain within a narrow range, and its rise may result from a raised formation, a reduced clearance, or multiple other factors [22,23]. Lactate levels have been found to help in the prediction or monitoring of the severity of pathologies that are critical in nature, like sepsis, cardiovascular emergencies, trauma, and burns [24]. The concentration of lactate ≥2.5 mmol/L is the definition of lactic acidosis [25]. Severe lactic acidosis occurs when the lactate level in the blood is >5 mmol/L, and the anion gap is ≥10 [26]. In DKA, hyperlactatemia is when lactate levels are >2 mmol/L [27].

Serum lactate levels are high in patients with DKA and may also be used in the emergency room to determine the severity of the patient’s condition [28,29]. A study conducted by Cetin et al. observed a positive correlation between serum lactate levels and the severity of DKA [30]. The study suggested that serum lactate levels can be used as a marker for the severity of this life-threatening emergency since there is very little time to intervene in critical cases of DKA. The study found the median lactate levels for mild, moderate, and severe DKA to be 1.9 mmol/L, 2.8 mmol/L, and 3.3 mmol/L, respectively. Serum lactate levels reflect a hypoperfusion of tissue and hypoxia and thus indicate the disease’s severity [4]. In the case of sepsis, there is also a rise in serum lactate levels, and a study conducted using the data from the Surviving Sepsis Campaign reported that the cut-off point for a serum lactate level of 4 mmol/L was linked to poor outcome in patients with sepsis [31,32]. Howell et al. noted a mortality rate of 28% in septic subjects with a serum lactate cut-off point of 4 mmol/L [33]. Another study by Shapiro et al. suggested that serum lactate levels may predict mortality in sepsis patients [34].

Another study conducted by Cox et al. found that 68% of patients with DKA had raised lactate levels, and 40% of the subjects had a cut-off value of more than 4 mmol/L [28]. High lactate levels did not result in an increased mortality in the study, and the mortality rate for subjects with serum lactate levels more than 4 mmol/L was 3.7% was due to the death of a subject suffering DKA with sepsis. They suggested that patients with DKA alone have a lower mortality rate than subjects with sepsis with similar serum lactate levels. Several other studies have found different values for the percentage of subjects with DKA having raised serum lactate levels, like 90% [29], 57% [35], and 67.9% [24].

Therefore, using serum lactate cut-off points can help recognize and monitor the severity of DKA and the possible presence of sepsis in the emergency room [30]. However, serum lactate alone may not be a specific diagnostic tool for DKA with infection since the raised lactate levels may have occurred due to fluid loss, microcirculation disorder, and stress intensity in DKA, even in the absence of infection [25,36]. Therefore, other diagnostic tools must also be included to determine if infection further complicates DKA.

### 6.2. Procalcitonin (PCT) in DKA

Another diagnostic tool that has been recommended for the diagnosis of DKA is PCT. It is a calcitonin precursor having no activity of hormone [4]. PCT has 116 amino acids, formed by neuroendocrine cells that include C cells of the thyroid gland [37]. PCT is a hormokine that gives rise to hormones; however, under inflammatory conditions, it may act as cytokine [38,39]. The release of PCT may be triggered by microbial toxins and cellular and humoral immune responses through interleukin-6, interleukin 1β, and tumor necrosis factor-α (TNF-α). Under septic conditions, parenchymal cells, like the liver, kidney, muscle, and adipocyte cells, can release hormokines [39].

In a healthy person, PCT formation does not occur in significant amounts. Conditions like surgery, trauma, shock, infection, and severe hyperglycemia can lead to a rise in PCT [4]. Large quantities of PCT may be formed when there is an extreme form of systemic inflammation. In severe systemic inflammation, PCT can markedly rise within two to twenty-four hours, which may persist until the patient recovers [40,41,42]. Since the half-life of PCT is longer than that of CRP and other acute phase reactants, that is, 22 to 26 h, it is advantageous to measure it [43]. However, PCT does not rise in viral infection due to the inhibition of TNF-α synthesis by α-interferon produced by macrophages [44]. Several studies have concluded that using PCT as a diagnostic tool can help properly use antibiotics in case of lower respiratory tract infections and sepsis [45,46]. PCT is beneficial for monitoring bacterial infection and may be a potential diagnostic tool for the differential diagnosis of systemic inflammatory response syndrome [47,48].

Several studies have reported DKA to increase the production of cytokines [18,19,49]. Hyperglycemia also leads to a rise in cytokine formation [50]. Therefore, DKA may cause an inflammatory environment and thus raise levels of PCT [51]. A study performed by Ivaska et al. noted a high level of PCT in children suffering from type 1 diabetes mellitus who developed DKA [52]. Two other studies have also observed an elevated PCT level in adults with type 1 diabetes mellitus who suffer from DKA [52,53]. Aksu et al. noted a drop in PCT levels after glycemia was normalized in subjects with acute hyperglycemic crises [54]. Similar findings were reported by another study, where there was a fall in PCT levels after the normalization of glycemia in the case of subjects with DKA without any proven infection [55]. Raised levels of PCT were also observed by Cipriano et al. in patients having DKA without any infection [56].

### 6.3. PCT Levels in Infection

**Figure 3:**
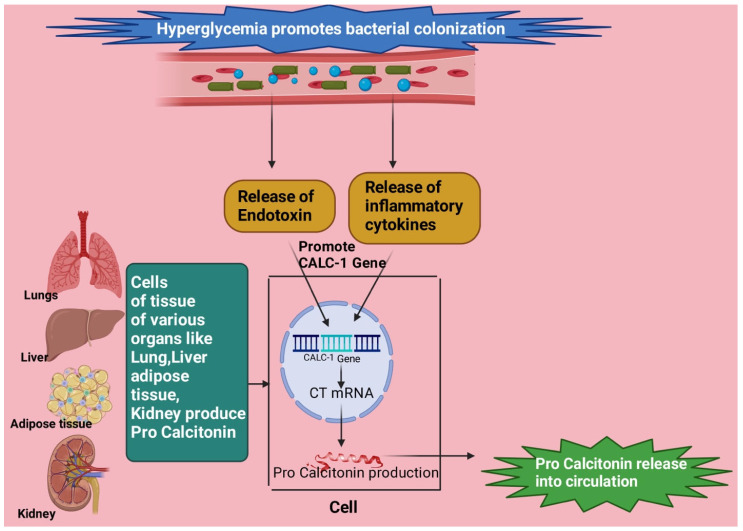
Endotoxin and inflammatory cytokines produced due to bacterial colonization in a hyperglycemic environment stimulate the CLAC-1 gene followed by CT mRNA formation, which causes the production of procalcitonin from tissues of organs like lung, liver, kidney, and adipose tissue. This figure has been drawn with the premium version of BioRender (https://biorender.com/ accessed on 23 June 2023), license number LJ25JGV5GM. Image credit: Rahnuma Ahmad.

The colonization of bacteria is promoted by hyperglycemic crisis and may lead to raised levels of PCT [57,58]. An induction of PCT by endotoxin and inflammatory cytokines occurs by enabling CALC-I gene transcription and CT mRNA expression in lungs, kidneys, liver, and adipose tissue [57,59] (Figure 3).

PCT has been noted to be of significant use for clinically diagnosing sepsis compared to other laboratory tests, and is associated with bacterial invasion severity [60,61,62]. PCT is also of prognostic value as it has a role in severe sepsis development, as noted in animal studies [44,63]. PCT has been of predictive value for recognizing mortality risk in critically ill individuals having infection [64]. PCT is not reduced with steroid and non-steroid anti-inflammatory drugs [65].

Hausfater et al. reported that those admitted to the hospital’s emergency department with fever had a PCT cut-off value of 0.2 ng/mL with a sensitivity and specificity for bacterial infection diagnosis of 0.77 and 0.59, respectively [66]. A meta-analysis performed by Wacker et al. reporting the clinical value and accuracy of PCT for diagnosing sepsis in patients who were critically ill found the sensitivity and specificity to be 0.77 and 0.79, respectively [67]. The PCT used for guiding the diagnosis of sepsis was summarized by Sager et al. They suggested that in critically ill patients, the patient was ‘likely’ to have a bacterial infection if the value of PCT was 0.5–1.0 ng/mL and ‘very likely’ if the value was above 1.0 ng/mL [68].

### 6.4. PCT in DKA Patients with Infection

A retrospective study was performed in France to seek PCT’s role in infection with DKA. The study selected proven cases of bacterial infections based on the positive cultures of bacteria in 102 DKA episodes and chose 20 cases as confirmed-bacterial-infection cases with DKA. Through a univariate analysis, PCT levels were significantly higher in individuals with proven bacterial infection than those without infective disorders. When multiple regression analysis was conducted with adjustments made for age, ketones, and insulin treatment, PCT levels higher than 1.44 ng/mL (OR 1.27 and 95% CI 1.04–1.63) were related to proven cases of bacterial infection. For the subjects without any confirmed bacterial infection, the PCT level on admission to the hospital and on the second day was 0.52 and 0.42 ng/mL, respectively. The sensitivity and specificity for PCT levels to differentiate patients with and without infection on admission to the hospital were 0.90 and 0.76, respectively. They suggested that a PCT level above 1.44 ng/mL on the day of hospital admission was of good value for predicting infection in patients with DKA [55].

Another study in Iraq found an elevated PCT level in patients suffering from diabetic foot infection compared to the group without infective disorders, the control group [69]. A study conducted in Indonesia found PCT to be higher in children suffering DKA with infection than infected children without DKA, with *p* < 0.001 [70].

Since several stressful conditions may result in a raised PCT, caution must be applied while using PCT as a diagnostic tool for DKA with infection. Since DKA gives rise to hypovolemia and dehydration, PCT adjusted for hypovolemia may help obtain a more precise outcome that can help diagnose DKA with infection in the emergency room [55].

### 6.5. Procalcitonin-to-Lactate Ratio (PLR) for Diagnosing DKA with Infection

From the above segment, it may be suggested that considering PCT alone for assessing DKA patients with infection may not be sufficient. Also, although high in DKA, lactate levels may be limited in diagnosing DKA with infective diseases [4,36]. In DKA, there is a severe loss of body fluid, which may lead to a decreased perfusion of tissue and lactate formation [25]. The intensity of stress-and-body-fluid volume may be assessed using lactate concentration in DKA [4,71]. Therefore, finding the ratio between procalcitonin to lactate may be of significant value as a diagnostic tool since, in this case, the procalcitonin level is adjusted for the induced stress and hypovolemia in DKA. A study found that the PLR threshold level of >0.438 may be considered for identifying infection in its early stages in individuals who develop DKA [4]. A study performed in China reported that the PLR in subjects with DKA and infection was 0.25 ± 0.11%, which was significantly more than that of the control group (*p* < 0.05). Also, the sensitivity and specificity of PLR for diagnosing DKA with infection were high, at 84.46% and 87.23%, respectively [72].

This novel factor PLR may be of high diagnostic value in both types of diabetes mellitus, and further studies may help establish this factor as an effective tool for diagnosing DKA with infection in the emergency setting.

### 6.6. Leucocyte Count for DKA with Infection

DKA raises systemic inflammation and oxidative stress [73,74,75]. Acidosis also causes platelet function downregulation and promotes neutrophil activity [17,76,77,78,79,80]. There is also the activation of T-lymphocytes [17,76,77,78,79,80,81]. The presence of infection also creates an environment of inflammation with the production of cytokines and oxidative stress [82].

Several studies have found a change in white blood cell count in DKA. A study by Karavanaki observed that blood leukocyte count was higher in DKA patients on arrival at the hospital, which was 15.2 × 10^2^/mm^3^ [83]. Another study noted that a rise in leukocyte levels was higher in DKA patients, and that those with infection had significantly higher blood leukocyte levels (16,910/mm^3^) in comparison to patients suffering DKA without infection (10,310/mm^3^) [76]. The study conducted by Huang et al. found that the threshold value for leukocyte count was >10 × 10^9^/L for DKA with infection [4].

### 6.7. Neutrophil-to-Lymphocyte Ratio (NLR) in DKA Patients

Among the fractions of white blood cells, attention has been drawn in recent years to NLR. A correlation exists between inflammation-related diseases like type 2 diabetes, neoplasm, obesity, hypertension, and atherosclerosis [84,85,86,87,88,89,90]. NLR shows adaptive and innate immune response functions [91,92,93,94]. Several studies have found an increased NLR in patients with DKA [82,95,96,97]. In a study, the median NLR in patients without ketoacidosis was 1.11; 0.80–1.80. In mild cases of DKA, it was 1.58; 1.17–1.93. In moderate cases of DKA value, it was 3.71; 1.98–4.85. In severe cases, NLR median value was 5.77; 4.04–9.63 [95]. NLR may be a possible predictor of systemic inflammation severity in DKA [98]. In DKA, the rise in NLR indicates an increase in neutrophil count and a decrease in the count of lymphocytes. This fall in lymphocyte count may be due to the damage of peripheral lymphocyte DNA by the reactive oxygen species promoted by an acute hyperglycemic state [98,99,100]. Similarly, sepsis also causes a marked rise in NLR by stimulating the apoptosis of lymphocytes and promoting neutrophils [101].

However, NLR may not be of good predictive value for infection in DKA since the stressful nature of DKA already raises NLR. A cut-off value of 10 is usually considered to be weakly linked to the presence of sepsis, and the higher the value above 10, the more the possibility of infection. Thus, NLR may help distinguish patients suffering from severe systemic inflammation from those with milder illnesses [102].

### 6.8. C Reactive Protein (CRP) and Interleukin-6 in DKA with Infection

The overexpression of acute inflammation can be detected by measuring CRP levels and interleukin-6. The critical phase proteins are released in systemic inflammatory conditions. A study conducted by Gogos et al. found a significantly higher CRP level (*p* < 0.001) and interleukin-6 (*p* < 0.0001) among patients suffering from DKA with infection when compared to those without infection [103]. Hoffman et al. found a significant rise in almost all the cytokines at the time of admission in patients with DKA. Neutrophil percentage, leukocyte count, and CRP in patients with DKA were raised compared to those without infection [19]. The study by Huang et al. also found similar results for raised leucocyte, neutrophil percentage, and CRP [4].

### 6.9. Body Temperature in DKA

The presence of fever may be a clinical marker for DKA with infection. Some studies reported a higher body temperature in patients having DKA with infection [55,103]. Gogos et al. found a body temperature of >38.5 °C in DKA patients with infection [103]. Blanchard et al. observed that the body temperature in patients without bacterial infection ranged between 32.9 °C and 38.7 °C since the patient was admitted. The thermoregulatory function may be impaired in patients with diabetes [55,104].

### 6.10. Combining Several Biomarkers to Make a Probable Diagnosis of DKA with Infection

**Figure 4:**
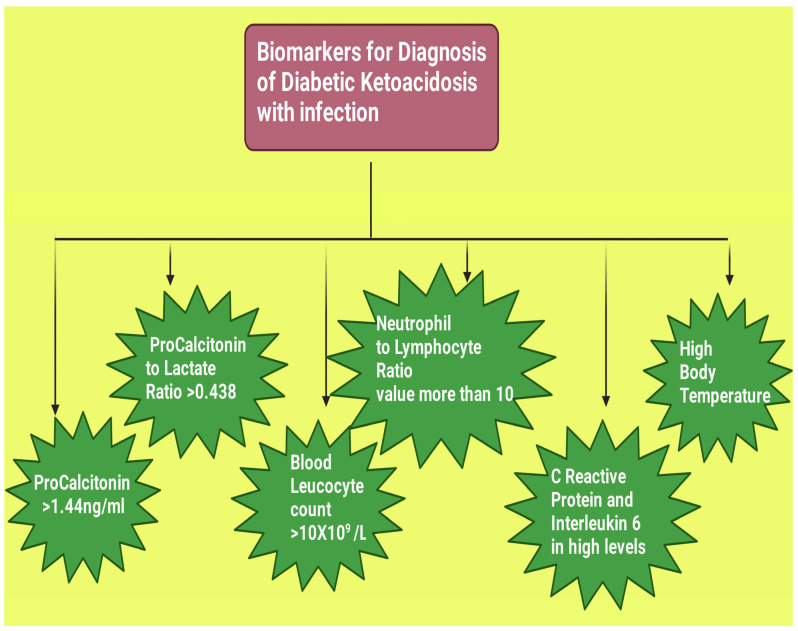
Road map for the various biomarkers for diagnosing diabetic ketoacidosis with infection. This figure has been drawn with the premium version of BioRender (https://biorender.com/ accessed on 23 June 2023), license number CH25MAR9Y4. Image credit: Rahnuma Ahmad.

The abovementioned biomarkers with their possible values in DKA patients with infection have been summarized (Table 1, Figure 4). Combining several of these biomarkers such as the presence of fever with PCT may aid in making a more specific diagnosis. Blanchard et al. noted the presence of both signs in patients with infection and DKA at the time of admission. In patients without infection, there was a near correction of PCT levels and white blood cell count following insulin administration and glycemia correction. However, in patients having DKA with infection, fever episodes continued to occur along with high PCT, white blood cells, neutrophil count, and NLR after the correction of glycemia. Thus, they suggested that the infection status can be determined based on PCT levels and fever presence on admission to the Department of Emergency. After correcting glycemia, antibiotic use (if already started) can be reassessed depending on the altered markers [55]. Huang et al. suggested from their study findings that leucocyte counts with a threshold value of >10 × 10^9^/L and PLR with a threshold value of >0.438 can help determine infection in its early stages in DKA patients [4].

### 6.11. Limitations of this Paper

The following were some limitations of the study. (i) This study is a narrative review, so there was no effort to perform any meta-analyses. Additionally, the number of total articles screened was not recorded, and only those discussed are included in the reference list. (ii) Studies in languages other than English could not be included. (iii) Articles that need to be accessed through institutional access could not be accessed.

## 7. Conclusions

In recent years, several studies have been carried out on different biomarkers to make the distinction between DKA with and without infection [4,30,52,55,76]. Infection may be detected early in DKA patients by using biomarkers like procalcitonin, serum lactate, procalcitonin/lactate ratio, white blood cell count, neutrophil/lymphocyte ratio, and C reactive protein. The values of these biomarkers were found to be higher in patients suffering from DKA with infection when compared to DKA patients without infection [4,55]. Also, these high values often persist in DKA patients with infection even after the correction of glycemia [55]. Several of these biomarkers need to be checked when the patient is admitted to the emergency department to determine whether DKA is further complicated with infection. There is limited data for the procalcitonin-to-lactate ratio and neutrophil-to-lymphocyte ratio, which are novel biomarkers that require further research. Awareness regarding the diagnostic tools for diagnosing this medical emergency may help physicians to provide proper management early. If the presence of infection is properly diagnosed, then antibiotics may be used appropriately and antibiotic resistance can be avoided.

## 8. Recommendation

We recommend further thorough research on the various markers, including cytokines and possibly other pathways that can result in more confirmatory diagnostic tests for DKA that are further complicated with infection. More research is needed to distinguish between DKA with and without infection. There is also a requirement to find the molecular pathways that may be targeted for the diagnosis and management of this complication and reduce the mortality rate further.

## 9. Article Highlights

Diabetes mellitus is a global health problem that has several complications, and diabetic ketoacidosis is an acute complication that requires hospitalization for early diagnosis and management. Several biomarkers exist for diagnosing DKA. Infection is a common trigger for diabetic ketoacidosis, and DKA patients who suffer from infection have a higher mortality rate. If a bacterial infection is present, it is imperative to administer antibiotics immediately to the patient. However, a bacterial culture report (a confirmatory test for bacterial infection) may take up to 24 h. Studies have been carried out to diagnose and distinguish between DKA with and without infection. Several biomarkers have been highlighted from such studies, such as serum lactate, procalcitonin, procalcitonin/lactate ratio, white blood cell count, and neutrophil/lymphocyte ratio. The cytokines and other mediators may overlap since DKA and infection lead to systemic inflammation. Therefore, upon comparing these mediators between DKA with and without infection, studies have found higher levels in DKA with infection compared to DKA without infection. Several of these biomarkers can be determined together to come to an approximate conclusion regarding the presence or absence of infection in DKA patients in the emergency setting. Researchers need to perform more studies to find more molecular pathways that may be targeted for diagnosing DKA with infection immediately with precision.

## Figures and Tables

**Table 1:** Illustrating the biomarkers for DKA with infection.

Biomarkers	Values that May Indicate DKA with Infection
Procalcitonin (PCT)	PCT level above 1.44 ng/mL on the day of hospital admission is of good predictive value for infection in patients with DKA [55].
2. Procalcitonin-to-lactate ratio (PLR)	PLR threshold level of >0.438 may be considered for identification of infection in its early stages in individuals who develop DKA [4].
3. Blood leucocyte count	The threshold value for the leukocyte count of >10 × 10^9^/L may be considered for DKA with infection [4].
4. Neutrophil-to-lymphocyte ratio (NLR)	A cut-off value of 10 is usually considered to weakly link to the presence of sepsis, and the higher the value above 10, the more the possibility of a presence of infection [102].
5. C reactive protein (CRP) and interleukin-6	Study conducted by Gogos et al. found a significantly higher CRP level (*p* < 0.001) and interleukin-6 (*p* < 0.0001) among patients suffering from DKA with infection when compared to those without infection [103].
6. Body temperature	Gogos et al. found a body temperature of >38.5 °C in DKA patients with infection [103].
